# Age differences in the use of serving size information on food labels: numeracy or attention?

**DOI:** 10.1017/S1368980016003219

**Published:** 2016-12-27

**Authors:** Lisa M Soederberg Miller, Elizabeth Applegate, Laurel A Beckett, Machelle D Wilson, Tanja N Gibson

**Affiliations:** 1 Department of Human Ecology, University of California, Davis, One Shields Avenue, Davis, CA 95616, USA; 2 Department of Public Health Sciences, University of California, Davis, Davis, CA, USA; 3 Department of Nutrition, University of California, Davis, Davis, CA, USA

**Keywords:** Nutrition label use, Food choice, Serving size information, Healthier choices

## Abstract

**Objective:**

The ability to use serving size information on food labels is important for managing age-related chronic conditions such as diabetes, obesity and cancer. Past research suggests that older adults are at risk for failing to accurately use this portion of the food label due to numeracy skills. However, the extent to which older adults pay attention to serving size information on packages is unclear. We compared the effects of numeracy and attention on age differences in accurate use of serving size information while individuals evaluated product healthfulness.

**Design:**

Accuracy and attention were assessed across two tasks in which participants compared nutrition labels of two products to determine which was more healthful if they were to consume the entire package. Participants’ eye movements were monitored as a measure of attention while they compared two products presented side-by-side on a computer screen. Numeracy as well as food label habits and nutrition knowledge were assessed using questionnaires.

**Setting:**

Sacramento area, California, USA, 2013–2014.

**Subjects:**

Stratified sample of 358 adults, aged 20–78 years.

**Results:**

Accuracy declined with age among those older adults who paid less attention to serving size information. Although numeracy, nutrition knowledge and self-reported food label use supported accuracy, these factors did not influence age differences in accuracy.

**Conclusions:**

The data suggest that older adults are less accurate than younger adults in their use of serving size information. Age differences appear to be more related to lack of attention to serving size information than to numeracy skills.

The US Centers for Disease Control and Prevention reports that 80 % of older adults have one or more chronic conditions^(^ ^1^ ^)^ and many of these chronic conditions are related to diet^(^ ^2^ ^)^. Serving size information appears within the nutrition facts panel on most packaged foods and provides important information for adhering to a healthful diet^(^ ^3^ ^–^ ^5^ ^)^. Although communication of serving size information is important for adults of all ages, age-related chronic conditions often require dietary modifications, which make it even more important for older adults to use information on food labels^(^ ^6^ ^)^. Given age-related changes in cognitive abilities^(^ ^7^ ^)^, older adults may be particularly at risk for failing to understand complex information on nutrition labels.

Serving size information is critical for accurately interpreting the nutrition information provided on the label, which is typically presented for a single serving of the product. Estimates vary, however, regarding the percentage of individuals who report using the serving size area of the food label. For example, serving size was among the top three areas of the food label used by US college students who reported use of specific areas (roughly a third of the sample)^(^ ^8^ ^)^. In a study in Malaysia, 60 % of college students rated serving size as the most commonly used area of the label^(^ ^9^ ^)^. The evidence surrounding older adults’ self-reported use of serving size information is scant, but findings suggest that older adults may use this area of the food label slightly more than do younger adults. In a representative study conducted in the USA, Olberding *et al*.^(^ ^10^ ^)^ found that 49 % of older adults and 40 % of younger adults reported using serving size information, which is somewhat lower than the 64 % of older and 52 % of younger adults who reported using the nutrition facts area below it.

The self-reported data reviewed above may reflect the challenges associated with interpreting serving size information. Research using objective or tested use of nutrition labels is consistent with this notion and shows that serving size information is difficult for most individuals to understand^(^ ^5^ ^)^. For example, 94 % and 76 % of college students were unable to determine the number of calories per container of bread and biscuits, respectively, using a nutrition label with more than one serving per package^(^ ^9^ ^)^. Similarly, in a study of adults aged 18–60 years, Pelletier *et al*.^(^ ^11^ ^)^ found that 86 % were unable to determine the number of calories per snack package when packages had more than one serving per container. Similar findings were reported in an online survey in which 83 % of adults (aged 18 to over 45 years) were unable to use a standard format nutrition label to identify the number of calories in a soda bottle with more than one serving per bottle^(^ ^12^ ^)^. In an online sample of adults (mean age 46 years), researchers presented single- or dual-column nutrition labels with the per-container information provided alongside the per-serving information^(^ ^13^ ^)^. Results showed that performance was higher for single-serving questions and products with dual-column formats, suggesting that the number of servings per container was not consulted or, if consulted, was not understood.

Although informative, these studies did not address older adults’ ability to use serving size information. Lubman *et al*.^(^ ^14^ ^)^ examined Russian immigrants’ ability to interpret a nutrition label from a two-serving bottle of milk, looking at age differences within a limited range. They found that 58 % of those in the younger group (aged 18–35 years) and 14 % of those in the older group (aged 36 years or older) were considered high scorers. Using a wider age range, Rothman and colleagues^(^ ^15^ ^)^ asked people, aged 18–80 years, to calculate the amount of carbohydrates in a 20-oz soda bottle with 2·5 servings. Performance was low overall, with 68 % of people unable to correctly answer the question, and total scores were lower for older (65 years or older) relative to younger adults (under 65 years of age)^(^ ^15^ ^)^. Importantly, the most common errors were misapplication of serving size and servings per container, confusion from other material on the food label, and incorrect calculations^(^ ^15^ ^)^. Mistakes were not examined by age group however, making it unclear why older adults had more difficulty than did younger adults.

The literature thus far suggests that serving size information is challenging to use, particularly among older adults. However, it is less clear why this information is difficult for older adults to use. Numeracy skills (ability to perform calculations) are one possibility, but another possibility is that individuals fail to notice the number of servings per container. A recent study showed that serving size information significantly reduced candy intake within a sample of 17–25-year-olds, but only among those who reported noticing the serving size recommendation^(^ ^16^ ^)^. In the current study, we characterized the change in accuracy with age. We then examined the strength of evidence for the competing hypotheses that numeracy *v*. attention to serving size information is a better predictor of accurate use of serving size information, with specific attention to how their effects on accuracy change with age.

## Methods

We examined attention to, and understanding of, serving size information within a sample of adults aged 20–80 years. In each trial, participants compared two brands of the same product on a computer screen to determine which was more healthful if they consumed the entire package. As shown in Table 1, we varied the task assigned to the participant (two versions), and within each task, a trial had either consistent or inconsistent nutrition information. In Task 1 (top left side of Table 1), participants were given no information regarding which specific nutrients to consider in their decision making. In Task 2 (top right side of Table 1), administered two weeks later, participants saw the same product pairs, but this time they were asked which food was more healthful specifically in terms of sodium or saturated fat, if they were to consume the entire package. Both tasks required participants to interpret nutrition label information for the entire package; however, Task 2 was presumably easier because it required consideration of only one nutrient (whereas all nutrients were potentially relevant in Task 1). The second way we varied the trials was either to have the two products differ so that one was healthier both per serving and per package (consistent trials, bottom left of Table 1) or to have one product healthier per serving but less healthy per package (inconsistent trials, bottom right of Table 1). We measured eye tracking to assess attention to label information and we assessed numeracy skills in the context of nutrition. We used these measures to examine whether numeracy and/or attention accounted for differences in accurate use of serving size information on these tasks. We also assessed food label habits (self-reported frequency of food label use) and prior nutrition knowledge to control for their effects on accuracy.

**Table 1:** Description of tasks and trial manipulations used in the present study

Task type	Task 1		Task 2	
Instructions				
Select the product that is	Healthier across all nutrients		Healthier on target nutrient only	
Search type	Undirected (compare multiple nutrients)		Directed (compare one nutrient)	
Trials	8 inconsistent/ 2 consistent		8 inconsistent/ 2 consistent	
Trial type	Inconsistent		Consistent (control)	
Sample pairs	Brand A	Brand B	Brand A	Brand B
More healthy per serving	x		x	
More healthy per package		x	x	
Need to use of number of servings per package?	Yes		No	

Brand, correct answer and presentation side (left, right) were counterbalanced across trials.

### Sample

We used stratified cluster sampling to recruit 1891 households in the Sacramento area, California, USA in 2013–2014 with publicly available telephone numbers, stratified by a socio-economic status index based on average education and income of the zip code. One thousand two hundred and eighty-six individuals between the ages of 18 and 80 years who were primary food shoppers were contacted by telephone and 238 were excluded due to report of neurodegenerative disease, head trauma or stroke, or report of rarely or never buying groceries for their household. Three hundred and ninety-two individuals met the study criteria and agreed to participate in the study. The Short Test of Functional Health literacy in Adults (S-ToFHLA) was used to screen out individuals with poor health literacy skills; three individuals with inadequate health literacy (health literacy assessment measure S-ToFHLA≤22) were omitted from the analyses^(^ ^17^ ^)^. Due to poor quality eye-tracker calibration for thirty-four individuals, the final sample included 358 participants aged 20–78 years (mean age 49·9 years). The Institutional Review Board of the University of California, Davis approved the study, and free and informed written consent of participants was obtained.

### Materials and equipment

**Fig. 1:**
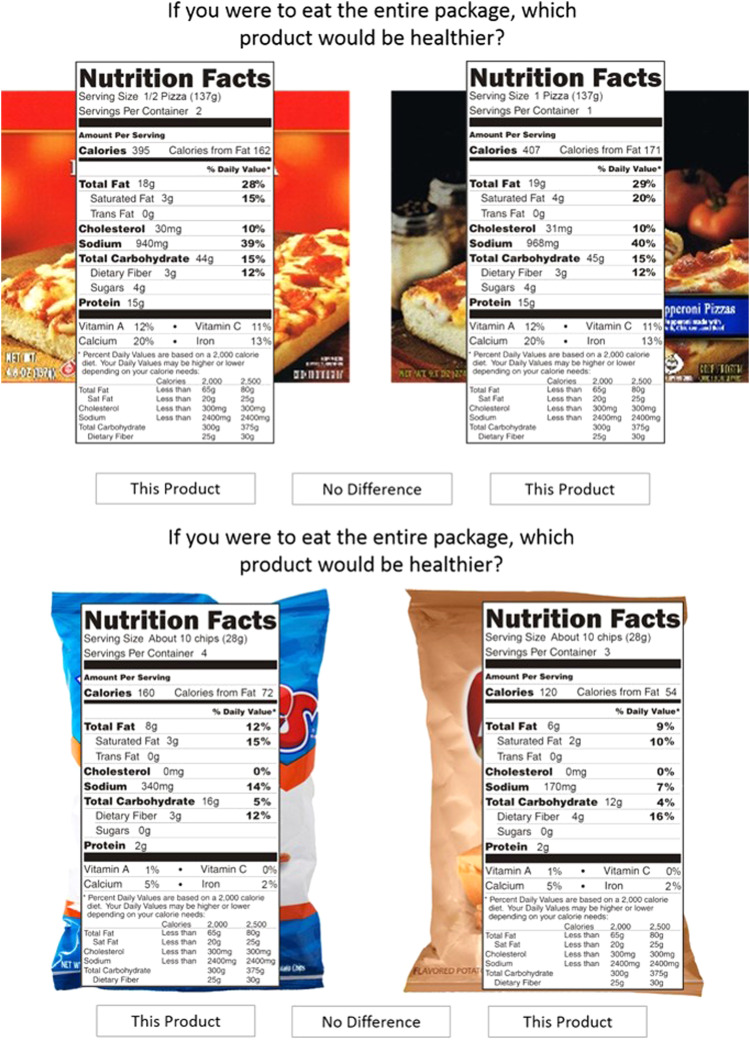
(colour online) Sample comparisons for inconsistent (top; per-serving and per-container information are inconsistent, so requires servings-per-container information for correct answer) and consistent (bottom; per-serving and per-container information are consistent with each other, so does not require servings-per-container information) trials

We selected ten pairs of commonly consumed foods: snacks and frozen pizzas, that were available in two popular brands. For both tasks, we manipulated the types of trials that were presented to be able to differentiate between the ability to use serving size information to determine healthfulness and the ability to determine healthfulness (regardless of serving size information). For eight pairs, we created a higher (Brand A) and lower (Brand B) servings-per-container version that differed slightly on a per-serving basis so that Brand A appeared to be the healthier choice if one were eating only one serving, but not if one were eating the entire package. These trials were referred to as ‘inconsistent’ trials because per-serving and per-container information lead to different answers and the individual had to use the servings-per-container information to arrive at the correct answer. Figure 1 shows examples of inconsistent and consistent trials. We also included two ‘consistent’ pairs in which the per-serving information and per-container information were consistent, both leading to the same answer, to assess the extent to which individuals were able to determine healthfulness without having to consider the number of servings per container. We used editing software to modify the image scans of actual package fronts and the nutrition labels (but keeping standard format as per Food and Drug Administration regulation 21 CR1 101.9(f)). Brand and servings-per-container combinations were counterbalanced so that each brand occurred equally often in each version and participants saw each brand version only one time. Table 1 summarizes both the trial and task manipulations.

Drawing on past work using eye-tracking methodology^(^ ^18^ ^,^ ^19^ ^)^, we assessed attention using EyeLink 1000, a video-based eye tracker that recorded the (*x*, *y*) coordinate position of the eye on the computer screen 1000 times per second, with an average accuracy of 0·25–0·5°. Coordinates that fell within regions of interest were examined for the amount of time spent viewing the information in those regions. Regions of interest for the present study were the serving size and servings per container section of the nutrition label. Attention was operationalized as the total dwell time on serving size information on the two nutrition labels for each trial, normed on their total viewing time for that trial, called proportion dwell time. The numeracy measure was modelled on Rothman *et al*.^(^ ^15^ ^)^ to assess the ability to manipulate quantitative information on nutrition labels. The measure was expanded from three items^(^ ^20^ ^)^ to seven items in the present study: (i) ‘How many grams of total fat are there in one container of this product?’; (ii) ‘How many grams of protein are there in one serving of this product?’; (iii) ‘How many servings of this product would one need to eat to get 100 % of the recommended daily value of calcium?’; (iv) ‘How many calories are there in one half of a container of this product?’; (v) ‘What percent of the recommended daily value of sodium is in one serving of this product?’; (vi) ‘How many grams of fibre are there in one half of a serving of this product?’; and (vii) ‘Roughly how many servings of this product would you need to get 100 % of the recommended daily value of iron?’ Nutrition knowledge was assessed using a twenty-five-item multiple-choice test of several sub-domains of knowledge such as dietary recommendations and sources of nutrients^(^ ^21^ ^)^. Scores on both measures were the total number correct. Self-reported food label use was assessed using the item: ‘I’d like you to think about the labels on many food products that list ingredients and provide nutrition and other information. When you buy a product for the first time, how often do you read this information?’ with a 5-point scale (5=‘always’, 1=‘never’)^(^ ^22^ ^)^.

### Procedure

Participants completed two serving size tasks, two weeks apart. Questionnaires including demographic questions, self-reported food label use, numeracy and nutrition knowledge were completed at the beginning of session 1. For both serving size tasks, participants viewed high-resolution images of the package fronts, presented side by side, on a wide-screen monitor while their eye movements were monitored. Image sizes were the same for both products, to resemble online shopping. After viewing the package front, participants clicked anywhere on the screen to proceed to the next screen, which displayed the nutrition label superimposed over its respective package front. Participants were instructed to click on the button below the more healthful product or, if there was no difference, to click on the button between the two products (labelled ‘no difference’). In Task 1, participants were asked to determine which product was more healthful if they consumed the entire package: ‘If you were to eat the entire box/bag/package, which product would be healthier?’ (see Fig. 1 for a sample comparison). Task 2 was designed to assess the effects of practice as well as more directed instructions on use of serving size information. In Task 2, the same product pairs were presented, however instructions were focused on one nutrient: ‘If you were to eat the entire box/bag, which one would have less saturated fat/sodium?’

### Statistical analysis

Since a key goal of the study was to gain a better understanding of how and why label use accuracy shifts with age, we summarized the characteristics of our sample descriptively (proportions for categorical variables, means and standard deviations for continuous variables) within each of three age groupings: less than 40 years, 40–60 years and over 60 years. We tested for significant differences of characteristics across age groups using *χ* ^2^ tests for categorical variables, ANOVA for continuous variables and mixed-effects ANOVA for average accuracy across repeated trials.

We used mixed-effects logistic regression models to test for associations between accuracy (odds of choosing the more nutritious option in a trial) and key personal characteristics (age in years as a continuous variable, education, income, nutrition knowledge, numeracy and self-reported food label use) and trial characteristics (inconsistency, task and how much attention was paid to the labels in that trial). We first tested the association of accuracy with each personal characteristic and each trial characteristic individually, in models with a single predictor (bivariate models). We set the reference level for age at 20 years and for attention, numeracy and nutrition knowledge at the maximum levels. This allowed us in subsequent multivariate models to use main effect estimates to characterize how accuracy differed for older, less attentive or less numerate participants compared with a reference group of young participants with high attention, numeracy and knowledge, and to use interactions to see what factors were associated with age differences in accuracy. Our first multivariate model included age×task and age×inconsistency interactions to see whether age differences, if found, were associated with trial characteristics (the more specific instructions in Task 2, or having inconsistent per-serving and per-package nutrition information). Our second multivariate model examined the possibility that the effects of attention and numeracy might change with age (two-way interactions); we also included interactions with age, inconsistency and the three-way interaction to see whether the effects of inconsistency on older adults’ accuracy depended on attention or numeracy. In the second multivariate model we controlled only for the covariates that were significant in the first multivariate model. Analyses were performed using the statistical software package SAS^®^ version 9.4.

## Results

Overall accuracy was low (50–55 %) across all age groups (Table 2). The proportion of males and levels of education, income, attention, label use and nutrition knowledge generally increased with age. However, numeracy did not differ significantly (*P*=0·58) with age.

**Table 2:** Sample characteristics by age; adults (*n* 358) aged 20–78 years, Sacramento area, California, USA, 2013–2014

	Age (years)				
Variable (% overall)	<40 (35 %)	40–60 (26 %)	>60 (39 %)	*P*	
Education				<0·001*	
≤High school (8 %)	8	11	5		
Some college (64 %)	71	69	53		
College graduate (28 %)	21	20	42		
Annual income ($US)				<0·001*	
≤34 999 (27 %)	41	22	19		
35 000–99 999 (51 %)	46	55	53		
100 000+ (23 %)	13	23	28		
Nutrition knowledge				0·021*	
<14 (18 %)	24	20	12		
14–19 (60 %)	60	61	59		
19+ (22 %)	16	19	29		
Sex					
(60 % female overall)	71	62	51	0·005*	
Self-reported food label use				0·034*	
At least some use (73 %)	69	67	80		
Rarely or never use (27 %)	31	33	20		
Attention					
Mean proportion dwell time	0·055	0·043	0·034	<0·001†	
sd	0·027	0·025	0·022		
Attention					
Mean proportion dwell time				<0·001*	
Low attention: ≤0·014	11	23	37		
Medium attention: 0·014–0·066	60	63	56		
High attention: >0·066	29	14	7		
Numeracy					
Mean	4·6	4·2	4·5	0·18	
sd	1·9	1·9	1·9		
Numeracy				0·58*	
≤3 (32 %)	28	36	31		
4–5 (34 %)	34	34	33		
6+ (34 %)	38	30	36		
				Age‡	Task‡
Accuracy					
Task 1 (Mean)	53	55	52	0·34	0·025
sd	17	17	15		
Task 2 (Mean)	52	51	50	0·63	
sd	22	22	19		

* Using *χ* ^2^ test.

† Using ANOVA.

‡ Using repeated-measures ANOVA.

The bivariate associations between accuracy and person-level and trial-level characteristics, not adjusted for any other covariates, are shown in Table 3. On average, accuracy was higher for participants who were younger, better educated, had more nutrition knowledge and had higher numeracy; accuracy was lower for those who reported reading labels more. Participants were more accurate in the second task (with more specific instructions) and in trials with consistent labels, and more accurate when paying closer attention to the labels.

**Table 3:** Bivariate associations of characteristics of participants and trials with accuracy, not adjusted for covariates, from mixed-effects logistic regression; adults (*n* 358) aged 20–78 years, Sacramento area, California, USA, 2013–2014

	Estimated effects on log odds of correct answer		
Predictor (reference category or units of measurement)	Estimate	se	P
Nutrition knowledge (difference from maximum)	0·16	0·02	<0·001
Income, low (reference=medium)	–1·04	0·18	<0·001
Income, high (reference=medium)	0·50	0·16	0·01
Education, high school (reference=college graduate)	–1·16	0·29	<0·001
Education, some college (reference=college graduate)	–0·43	0·17	0·01
Sex (male)	–0·26	0·16	0·06
Self-reported food label use	0·21	0·08	0·005
Task (reference=^1^)	0·15	0·06	0·009
Age (years since age 20)	–0·01	0·005	0·005
Inconsistency (reference=consistent)	–0·69	0·07	<0·001
Attention (difference from maximum)	9·70	0·95	<0·001
Numeracy (difference from maximum)	0·49	0·03	<0·001

The first multivariate model, presented in Table 4, examined the effects of participant characteristics and of inconsistency and task on the odds of correct answer. Regardless of age, higher nutrition knowledge (*P*=0·002) and higher numeracy (*P*<0·001) were associated with more accuracy, on average, as was being in the highest category of reported income (*P*=0·009, compared with medium income). Greater attention to the labels (longer viewing times) was also associated with greater accuracy, on average (*P*<0·001). The effects of age and trial characteristics, however, showed a more complex pattern. Young participants showed no significant difference in accuracy between Tasks 1 and 2 (task main effect, *P*=0·57) or between inconsistent and consistent trials (inconsistency main effect, *P*=0·13). Older age was not associated with a significant difference in accuracy for Task 1 or for consistent labels (age main effect, *P*=0·16). However, the older the participant, the lower the accuracy for inconsistent trials compared with consistent trials (age×inconsistency interaction, *P*<0·001). In addition, older adults were more accurate for Task 2, the more specific assignment, than for Task 1 (age×task interaction, *P*=0·023). Thus older adults on average showed a decrease in accuracy, as noted in Table 2, but that deficit was specific to trials requiring the use of serving size information (inconsistent trials) and those with less specific directions (Task 1).

**Table 4:** Preliminary logistic regression model: effects of characteristics of participants and trials on log odds of correct answer; adults (*n* 358) aged 20–78 years, Sacramento area, California, USA, 2013–2014

	Solutions for fixed effects (estimated log odds for correct answer)		
Effect (reference category or units of measurement)	Estimate	se	*P*
Participant characteristics			
Age (years since age 20)	0·008	0·006	0·16
Nutrition knowledge (difference from maximum)	0·07	0·02	0·002
Income, low (reference=medium)	0·07	0·15	0·65
Income, high (reference=medium)	0·41	0·16	0·009
Sex	0·06	0·13	0·64
Numeracy (difference from maximum)	0·41	0·04	<0·001
Self-reported food label use	0·02	0·06	0·71
Trial characteristics			
Task (reference=^1^)	–0·07	0·12	0·57
Inconsistency (reference=consistent)	0·23	0·15	0·13
Attention (difference from maximum)	8·70	0·95	<0·001
Interactions: age and trial characteristics			
Age×task	0·008	0·004	0·02
Age×inconsistency	–0·03	0·005	<0·001

Greater attention and higher numeracy were both strongly associated with greater accuracy overall (both *P*<0·001), as shown in Table 4, so we next examined whether either or both contributed specifically to increased accuracy for older adults on the more challenging trials (inconsistent trials and Task 1). As in our previous multivariate model, we found that greater nutrition knowledge (*P*<0·001) and high income (*P*=0·007) were associated with greater accuracy regardless of age, numeracy or trial type (Table 5). For participants with high numeracy, paying close attention, there was little difference in accuracy with age, at least for consistent trials in Task 1 (age main effect, *P*=0·63). For young participants, numeracy made little difference in accuracy (numeracy main effect, *P*=0·94).

**Table 5:** Logistic regression model: effects of characteristics of participants and trials on accuracy, and modification of age-related accuracy patterns by attention and numeracy; adults (*n* 358) aged 20–78 years, Sacramento area, California, USA, 2013–2014

	Solutions for fixed effects (estimated log odds for correct answer)		
Effect (reference category or units of measurement)	Estimate	se	*P*
Participant characteristics			
Age (years since age 20)	–0·01	0·02	0·63
Nutrition knowledge (difference from maximum)	0·07	0·02	<0·001
Income, low (reference=medium)	0·06	0·16	0·68
Income, high (reference=medium)	0·44	0·16	0·007
Numeracy (difference from maximum)	0·007	0·09	0·94
Trial characteristics			
Task (reference=^1^)	–0·08	0·12	0·49
Inconsistency (reference=consistent)	4·20	0·69	<0·001
Attention (difference from maximum)	–5·13	3·00	0·09
Interactions: age, numeracy and attention			
Age×attention	–0·08	0·10	0·44
Age×task	0·008	0·004	0·03
Age×numeracy	0·002	0·002	0·31
Age×inconsistency	0·04	0·02	0·11
Attention×inconsistency	15·00	3·50	<0·001
Numeracy×inconsistency	0·44	0·08	<0·001
Age×attention×inconsistency	0·27	0·12	0·02
Age×numeracy×inconsistency	0·002	0·002	0·51

The effects of age, attention and numeracy varied markedly, however, across different types of label-reading trials. Even for younger participants, inconsistency could pose a challenge. Young participants with high attention were, paradoxically, more accurate on inconsistent trials than on consistent trials (inconsistency main effect, *P*<0·001), regardless of task. Apparently, on these relatively easy trials, a quick glance at serving size information was sufficient for higher-performing younger adults to compare the products. Younger participants with higher numeracy were also more accurate on inconsistent trials than their counterparts with lower numeracy (numeracy×consistency interaction, *P*<0·001), and those paying closer attention were more accurate on the inconsistent trials (attention×inconsistency, *P*<0·001). Older participants were more accurate on the more specific Task 2 than on Task 1 (age×task, *P*=0·029), as in the previous analysis.

**Fig. 2:**
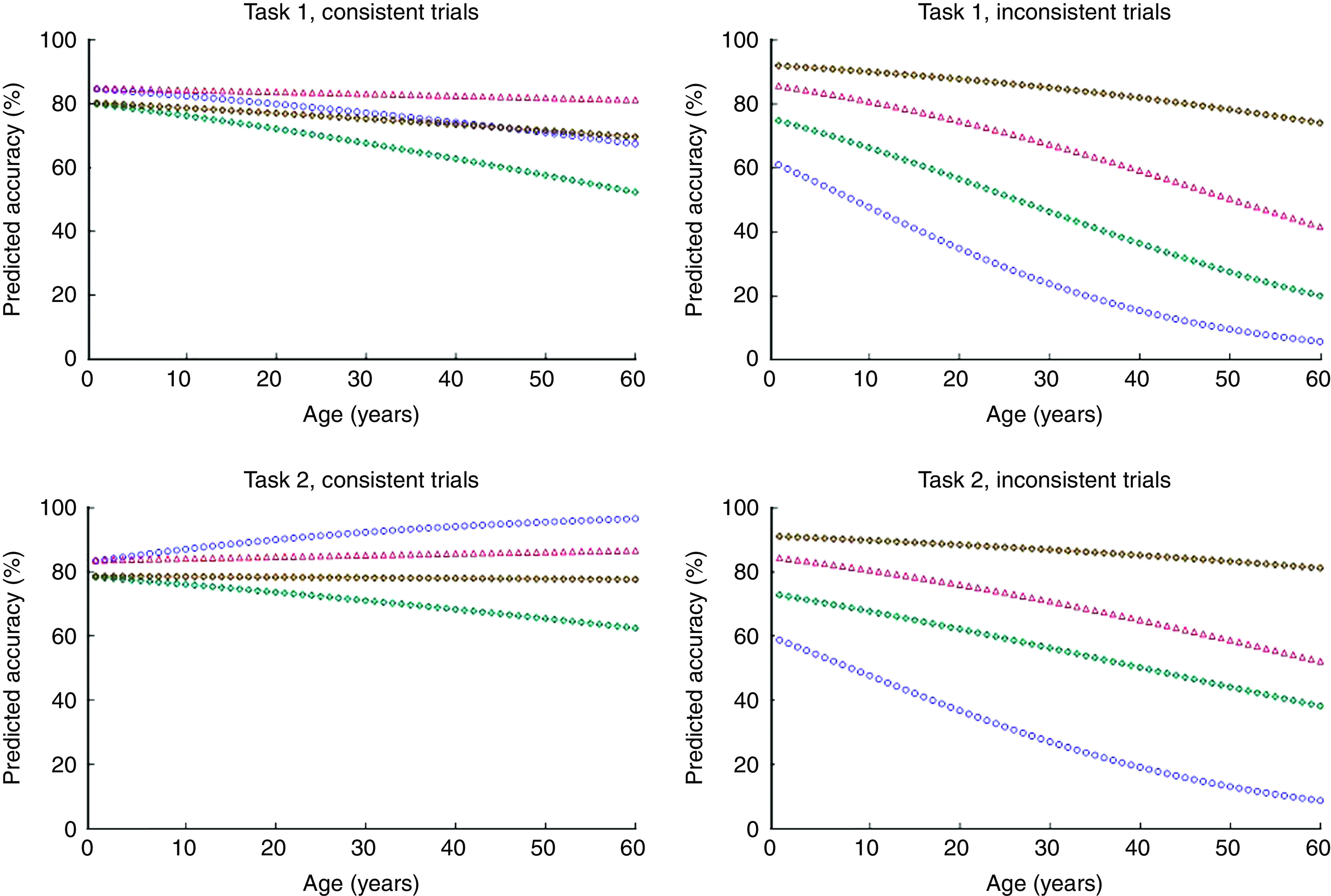
(colour online) Predicted values from attention model in Table 5 showing the association between age and accuracy for high and low levels of attention and numeracy (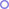, low attention, low numeracy; 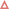, low attention, high numeracy; 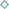, high attention, low numeracy; 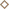, high attention, high numeracy) in Task 1 (top row) and Task 2 (bottom row), for consistent (left column) *v*. inconsistent trials (right column). Nutrition knowledge and income are set at high and median levels, respectively

The effects of attention and numeracy were most pronounced for older adults on trials requiring use of serving size information (inconsistent trials). Older adults paying close attention, and with high numeracy, did not show a significant difference in accuracy from younger people on the inconsistent trials (age×inconsistency, *P*=0·11). However, like younger adults, they showed a loss in accuracy for lower numeracy (numeracy×inconsistency interaction, *P*<0·001, not modified by age, see age×numeracy×inconsistency 3-way interaction, *P*=0·51). As shown in Fig. 2, older adults showed an even greater gain in accuracy than younger adults when they paid close attention in inconsistent trials (age×attention×inconsistency 3-way interaction, *P*=0·018).

## Discussion

Serving size information on nutrition labels is important for older adults seeking to manage diet-related chronic conditions. However, research suggests that older adults have difficulties understanding this information, perhaps due to numeracy skills^(^ ^4^ ^,^ ^15^ ^)^. The present study attempted to shed light on factors associated with accurate use of serving size information across adults of all ages and to identify problems that may be specific to older adults in particular.

Our results showed that accuracy in comparing nutrition labels can be very high even in older people, and even for more difficult comparisons where per-serving and per-package information is inconsistent. Accuracy is compromised for these trickier comparisons, however, by poor numeracy, regardless of age, and by poor attention, especially for older participants. Older participants also found it harder to make accurate comparisons when they were not given specific guidance about which nutrient should be the focus of their comparison. Nutrition knowledge was helpful across all ages, and higher-income participants were more accurate regardless of age.

The findings from the present study also showed that both numeracy and attention were important for accurate use of serving size information. The finding that numeracy was a strong predictor of accuracy suggests that numeracy skills support calculations required to translate per-serving information into per-container information. This finding aligns well with past research showing that numeracy skills are related to food label use tasks, including use of serving size information^(^ ^15^ ^)^. We also found that attention to serving size information significantly predicted accuracy such that the more attention individuals allocated to serving size information, the more accurate they were at using this information to determine which product was more healthful.

In addition to these findings, the data showed that older adults were less skilled at using serving size information to evaluate healthfulness relative to younger adults. On the other hand, when serving size information was unnecessary, younger and older adults were equally able to evaluate healthfulness. Thus, the findings suggest that older adults were more likely to base their decision on per-serving information, which led them to select the less healthful product on inconsistent pairs. Although older adults’ performance improved on the second task that directed them to consider only one nutrient, they were still more likely to use per-serving rather than per-container information, especially if they paid less attention.

Although the findings above do not rule out the possibility that failure to consider serving size information was driven by numeracy skills, we found that age declines in accuracy were not explained by numeracy. The strongest determinant of age differences in accuracy was attention to serving size information. Older adults who paid attention to serving size information were as accurate as younger adults. The finding that high-attention older adults performed well suggests that when older adults look at the number of servings per container, they are able to do the calculations required to arrive at the correct answer. Given that we controlled for numeracy skills, it does not appear to be the case that numeracy skills drove their attention to the number of servings per container. Rather, age declines in accurate use of serving size information appear to be due to a failure to consider the number of servings in a container of food.

These findings build on past work showing that older adults are less accurate than younger adults on nutrition label tasks that rely on manipulating numerical information^(^ ^15^ ^)^. The most common error made by participants in Rothman *et al*.’s study was not attempting to use serving size information or failure to use it accurately^(^ ^15^ ^)^. The present study builds on this by examining attention in addition to numeracy and determining whether either factor could account for age differences in use of serving size information. The data suggest that numeracy may reflect a general inability to use numerical information; however, the deficit was not unique to older adults.

The present study differs from past work in several ways. First, we included comparisons that did not require individuals to consider the number of servings per container (consistency) to determine whether nutrition information could be correctly interpreted without this added burden. Second, we examined whether practice together with directed instructions to consider only one nutrient influenced the accuracy using serving size information. Specifically, Task 1 required individuals to evaluate package healthfulness without explicit instruction regarding which nutrients to evaluate, whereas in Task 2, they were instructed to evaluate one particular nutrient. As expected, the second task was less difficult, but performance remained lower than we had expected, suggesting that practice together with limiting the number of nutrients to consider may not be sufficient to remove barriers associated with accurate use of serving size information. For older adults, the core problem appeared to be lack of consideration of multiple servings rather than too many nutrients to evaluate (calculate) at the package level or numeracy skills in general.

### Limitations

Although the study design had some strengths, such as use of popular brands to provide a realistic context, it also had some limitations. The study was correlational in nature, making it unclear if those who did not consult serving size information would have been able to use it accurately had they consulted it. Also, those who did not consult serving size information may have thought they would be confused by it. Indeed, consumers may avoid the food label altogether because the perceived difficulty of using the information could exceed the perceived benefits^(^ ^23^ ^)^. However, in the present study, we controlled for self-reported food label use as well as nutrition knowledge, which are related to skill and self-efficacy^(^ ^24^ ^,^ ^25^ ^)^, making this possibility less likely. Another possible limitation is that our numeracy measure was domain-specific, with items based on numerical information on food labels, making it unclear if a more generic measure would have shown similar results. Nevertheless, we found younger and older adults had similar numeracy scores which is consistent with past research that used a general assessment of numeracy^(^ ^15^ ^)^.

### Implications

The data presented here are consistent with the notion that serving size information on food packages is not readily seen or used by consumers. In its report on front-of-package nutrition labels, the Institute of Medicine called for improvements to serving size communication^(^ ^26^ ^)^. Specifically, serving size information should be prominently placed and formatted for ease of comprehension to help individuals place serving size information of a product within the context of other foods and beverages they consume^(^ ^26^ ^)^. Directive or semi-directive labelling systems^(^ ^27^ ^)^ that provide interpretation of the number of servings per container may be particularly helpful for older adults. Moreover, some evidence suggests that variability in how serving sizes are set across manufacturers can have the unintended consequence of increasing consumption on a per-package level^(^ ^28^ ^)^. Thus, individuals may also benefit from having nutrition labels that present serving size information standardized by weight, to ease interpretation^(^ ^28^ ^,^ ^29^ ^)^. In line with past work on younger adults^(^ ^13^ ^,^ ^30^ ^)^, findings from the present study also suggest that also individuals would benefit from label format changes that include detailed servings-per-container information, for example, a dual-column format. Finally, the findings suggest that there is a need, particularly among older adults, for training that increases awareness of how to use serving size and servings-per-container information to guide healthy food choices.
